# 3-(4-Chloro­phenyl­sulfon­yl)-2-methyl­naphtho[1,2-*b*]furan

**DOI:** 10.1107/S1600536808009215

**Published:** 2008-04-16

**Authors:** Hong Dae Choi, Pil Ja Seo, Byeng Wha Son, Uk Lee

**Affiliations:** a Department of Chemistry, Dongeui University, San 24 Kaya-dong, Busanjin-gu, Busan 614-714, Republic of Korea; b Department of Chemistry, Pukyong National University, 599-1 Daeyeon 3-dong, Nam-gu, Busan 608-737, Republic of Korea

## Abstract

The title compound, C_19_H_13_ClO_3_S, was prepared by the oxidation of 3-(4-chloro­phenyl­sulfan­yl)-2-methyl­naphtho[1,2-*b*]furan with 3-chloro­peroxy­benzoic acid. The 4-chloro­phenyl ring makes a dihedral angle of 68.59 (5)° with the plane of the naphthofuran fragment. The crystal structure is stabilized by π–π inter­actions between the benzene rings of neighbouring mol­ecules [centroid–centroid distance = 3.635 (3) Å], and by C—H⋯π inter­actions between a methyl H atom and the furan ring of an adjacent mol­ecule. In addition, the crystal structure exhibits inter­molecular C—H⋯O inter­actions.

## Related literature

For the crystal structures of similar 2-methyl­naphtho[1,2-*b*]furan derivatives, see: Choi *et al.* (2006 ▶, 2008 ▶).
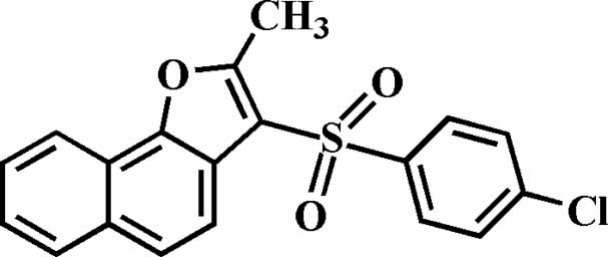


## Experimental

### 

#### Crystal data

C_19_H_13_ClO_3_S
*M* *_r_* = 356.80
Orthorhombic, 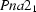

*a* = 8.1155 (3) Å
*b* = 18.6014 (7) Å
*c* = 10.8319 (4) Å
*V* = 1635.18 (11) Å^3^
*Z* = 4
Mo *K*α radiation
μ = 0.38 mm^−1^
*T* = 173 (2) K
0.60 × 0.40 × 0.40 mm

#### Data collection

Bruker SMART CCD diffractometer
Absorption correction: multi-scan (*SADABS*; Sheldrick, 1999 ▶) *T*_min_ = 0.842, *T*_max_ = 0.857
9543 measured reflections
2611 independent reflections
2532 reflections with *I* > 2σ(*I*)
*R*_int_ = 0.015

#### Refinement

*R*[*F* ^2^ > 2σ(*F* ^2^)] = 0.024
*wR*(*F* ^2^) = 0.066
*S* = 1.06
2611 reflections
218 parameters
1 restraint
H-atom parameters constrained
Δρ_max_ = 0.26 e Å^−3^
Δρ_min_ = −0.20 e Å^−3^
Absolute structure: Flack (1983 ▶), 728 Freidel pairs
Flack parameter: −0.01 (5)

### 

Data collection: *SMART* (Bruker, 2001 ▶); cell refinement: *SAINT* (Bruker, 2001 ▶); data reduction: *SAINT*; program(s) used to solve structure: *SHELXS97* (Sheldrick, 2008 ▶); program(s) used to refine structure: *SHELXL97* (Sheldrick, 2008 ▶); molecular graphics: *ORTEP-3* (Farrugia, 1997 ▶) and *DIAMOND* (Brandenburg, 1998 ▶); software used to prepare material for publication: *SHELXL97*.

## Supplementary Material

Crystal structure: contains datablocks global, I. DOI: 10.1107/S1600536808009215/at2553sup1.cif

Structure factors: contains datablocks I. DOI: 10.1107/S1600536808009215/at2553Isup2.hkl

Additional supplementary materials:  crystallographic information; 3D view; checkCIF report

## Figures and Tables

**Table 1:** Hydrogen-bond geometry (Å, °) *Cg*3 is the centroid of the O1/C12/C1/C2/C11 furan ring.

*D*—H⋯*A*	*D*—H	H⋯*A*	*D*⋯*A*	*D*—H⋯*A*
C19—H19*A*⋯*Cg*3^i^	0.98	2.89	3.488 (3)	120
C8—H8⋯O2^ii^	0.95	2.48	3.428 (2)	173

Symmetry codes: (i) 
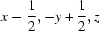
; (ii) 
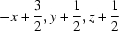
.

**Fig. 1.**
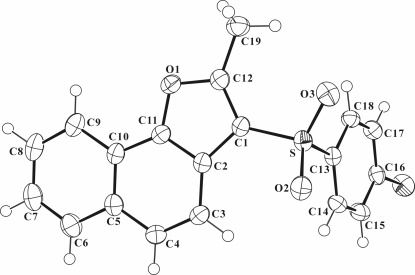
The molecular structure of the title compound, showing displacement ellipsoides drawn at the 50% probability level.

**Fig. 2.**
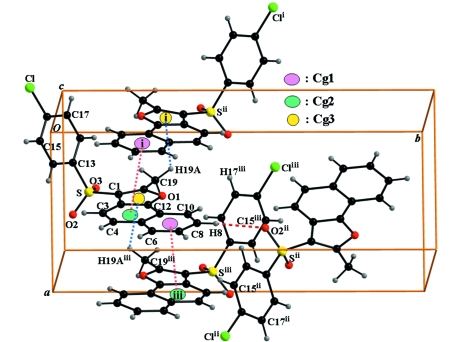
π—π, C—H···π and C—H···O interactions (dotted lines) in the structure of the title compound. Cg denotes the ring centroids. [Symmetry code: (i) x-1/2, -y+1/2, z; (ii) -x+3/2, y+1/2, z+1/2.]

## supplementary crystallographic information

### Comment

This work is related to earlier communications on the synthesis and structure of 2-methylnaphtho[1,2-b]furan analogues, *viz. *2-methyl-3-(methylsulfinyl) naphtho[1,2-b]furan (Choi *et al.*, 2006) and 2-methyl-3-(phenylsulfonyl) naphtho[1,2-b]furan (Choi *et al.*, 2008). Herein we report the molecular and crystal structure of the title compound, 3-(4-chlorophenylsulfonyl)-2-methylnaphtho[1,2-b]furan (Fig. 1).

The naphthofuran unit is essentially planar, with a mean deviation of 0.007 Å from the least-squares plane defined by the thirteen constituent atoms. The 4-chlorophenyl ring (C13-C18) makes a dihedral angle of 68.59 (5)° with the plane of the naphthofuran fragment. The crystal packing (Fig. 2) is stabilized by aromatic π—π stacking interactions between the benzene rings from the adjacent molecules. The Cg1···Cg2^iii^ distance is 3.635 (3) Å (Cg1 and Cg2 are the centroids of the C5-C10 benzene ring and the C2/C3/C4/C5/C10/C11 benzene ring, respectively, symmetry code as in Fig. 2). The molecular packing is further stabilized by C—H···π interactions between a methyl H atom and the furan ring of the naphthofuran unit, with a C19—H19A···Cg3^i^ separation of 2.89 Å (Fig. 2 and Table 1; Cg3 is the centroid of the O1/C12/C1/C2/C11 furan ring; symmetry code as in Fig. 2). Additionally, intermolecular C—H···O interactions in the structure were observed (Fig. 2 and Table 1; symmetry code as in Fig. 2)

### Experimental

3-Chloroperoxybenzoic acid (77%, 336 mg, 1.5 mmol) was added in small portions to a stirred solution of 3-(4-chlorophenylsulfanyl)-2-methylnaphtho[1,2-b]furan (227 mg, 0.7 mmol) in dichloromethane (30 ml) at 273 K. After being stirred at room temperature for 4 h, the mixture was washed with saturated sodium bicarbonate solution and the organic layer was separated, dried over magnesium sulfate, filtered and concentrated in vacuum. The residue was purified by column chromatography (hexane-ethyl acetate, 2 : 1 v/v) to afford the title compound as a colourless solid [yield 82 %, m.p. 427-428 K; R_f_ = 0.69 (hexane-ethyl acetate, 2 : 1 v/v)]. Single crystals suitable for X-ray diffraction were prepared by slow evaporation of a solution of the title compound in acetone at room temperature. Spectroscopic analysis: ^1^H NMR (CDCl_3_, 400 MHz) δ 2.91 (s, 3H), 7.45-7.64 (m, 4H), 7.75 (d, J = 8.44 Hz, 1H), 7.90-8.02 (m, 4H), 8.21 (d, J = 8.04 Hz, 1H); EI-MS 358 [M+2], 356 [M^+^].

### Refinement

All H atoms were geometrically positioned and refined using a riding model, with C—H = 0.95 Å for aromatic H atoms and 0.98 Å for methyl H atoms, respectively, and with *U*_iso_(H) = 1.2U_eq_(C) for aromatic and *U*_iso_(H) = 1.5U_eq_(C) for methyl H atoms.

### Crystal data

**Table:**

C_19_H_13_ClO_3_S	*D*_x_ = 1.449 Mg m^−^^3^
*M**_r_* = 356.80	Melting point = 427–428 K
Orthorhombic, *P**n**a*2_1_	Mo *K*α radiation λ = 0.71073 Å
Hall symbol: P 2c -2n	Cell parameters from 7325 reflections
*a* = 8.1155 (3) Å	θ = 2.2–28.2º
*b* = 18.6014 (7) Å	µ = 0.38 mm^−^^1^
*c* = 10.8319 (4) Å	*T* = 173 (2) K
*V* = 1635.18 (11) Å^3^	Block, colourless
*Z* = 4	0.60 × 0.40 × 0.40 mm
*F*_000_ = 736	

### Data collection

**Table:**

Bruker SMART CCD diffractometer	2611 independent reflections
Radiation source: fine-focus sealed tube	2532 reflections with *I* > 2σ(*I*)
Monochromator: graphite	*R*_int_ = 0.016
Detector resolution: 10.0 pixels mm^-1^	θ_max_ = 27.0º
*T* = 173(2) K	θ_min_ = 2.7º
φ and ω scans	*h* = −10→10
Absorption correction: multi-scan(SADABS; Sheldrick, 1999)	*k* = −23→23
*T*_min_ = 0.842, *T*_max_ = 0.857	*l* = −6→13
9543 measured reflections	

### Refinement

**Table:**

Refinement on *F*^2^	Hydrogen site location: difference Fourier map
Least-squares matrix: full	H-atom parameters constrained
*R*[*F*^2^ > 2σ(*F*^2^)] = 0.024	*w* = 1/[σ^2^(*F*_o_^2^) + (0.0417*P*)^2^ + 0.2762*P*] where *P* = (*F*_o_^2^ + 2*F*_c_^2^)/3
*wR*(*F*^2^) = 0.066	(Δ/σ)_max_ < 0.001
*S* = 1.06	Δρ_max_ = 0.26 e Å^−^^3^
2611 reflections	Δρ_min_ = −0.20 e Å^−^^3^
218 parameters	Extinction correction: none
1 restraint	Absolute structure: Flack (1983), 728 Freidel pairs
Primary atom site location: structure-invariant direct methods	Flack parameter: −0.01 (5)
Secondary atom site location: difference Fourier map	

### Special details

**Table:**

Geometry. All esds (except the esd in the dihedral angle between two l.s. planes) are estimated using the full covariance matrix. The cell esds are taken into account individually in the estimation of esds in distances, angles and torsion angles; correlations between esds in cell parameters are only used when they are defined by crystal symmetry. An approximate (isotropic) treatment of cell esds is used for estimating esds involving l.s. planes.
Refinement. Refinement of F^2^ against ALL reflections. The weighted R-factor wR and goodness of fit S are based on F^2^, conventional R-factors R are based on F, with F set to zero for negative F^2^. The threshold expression of F^2^ > 2sigma(F^2^) is used only for calculating R-factors(gt) etc. and is not relevant to the choice of reflections for refinement. R-factors based on F^2^ are statistically about twice as large as those based on F, and R- factors based on ALL data will be even larger.

### Fractional atomic coordinates and isotropic or equivalent isotropic displacement parameters (Å^2^)

**Table:**

	*x*	*y*	*z*	*U*_iso_*/*U*_eq_	
Cl	−0.09521 (6)	−0.07862 (3)	0.70659 (5)	0.04239 (14)	
S	0.49747 (5)	0.08522 (2)	0.47158 (5)	0.02560 (10)	
O1	0.55982 (14)	0.27683 (6)	0.61467 (14)	0.0294 (3)	
O2	0.64177 (14)	0.04043 (6)	0.48194 (16)	0.0336 (3)	
O3	0.44235 (16)	0.10742 (7)	0.35113 (14)	0.0344 (3)	
C1	0.5352 (2)	0.16153 (8)	0.55974 (18)	0.0247 (3)	
C2	0.62790 (19)	0.16344 (9)	0.67380 (18)	0.0256 (4)	
C3	0.7034 (2)	0.11205 (9)	0.75222 (19)	0.0292 (4)	
H3	0.6974	0.0621	0.7341	0.035*	
C4	0.7847 (2)	0.13619 (9)	0.85426 (19)	0.0305 (4)	
H4	0.8359	0.1022	0.9073	0.037*	
C5	0.7955 (2)	0.21145 (10)	0.88442 (19)	0.0302 (4)	
C6	0.8787 (2)	0.23535 (11)	0.9915 (2)	0.0386 (5)	
H6	0.9282	0.2014	1.0456	0.046*	
C7	0.8882 (3)	0.30743 (12)	1.0175 (2)	0.0449 (5)	
H7	0.9442	0.3229	1.0898	0.054*	
C8	0.8164 (3)	0.35875 (11)	0.9391 (2)	0.0421 (5)	
H8	0.8249	0.4084	0.9585	0.051*	
C9	0.7342 (2)	0.33765 (9)	0.8346 (2)	0.0347 (4)	
H9	0.6858	0.3725	0.7817	0.042*	
C10	0.7220 (2)	0.26328 (9)	0.80628 (19)	0.0280 (4)	
C11	0.63904 (19)	0.23541 (8)	0.7025 (2)	0.0261 (3)	
C12	0.4971 (2)	0.23071 (9)	0.5286 (2)	0.0280 (4)	
C13	0.3328 (2)	0.03991 (8)	0.54446 (17)	0.0244 (3)	
C14	0.3626 (2)	−0.01619 (9)	0.6257 (2)	0.0309 (4)	
H14	0.4725	−0.0296	0.6455	0.037*	
C15	0.2315 (2)	−0.05239 (10)	0.6775 (2)	0.0347 (4)	
H15	0.2495	−0.0909	0.7335	0.042*	
C16	0.0717 (2)	−0.03142 (10)	0.64619 (18)	0.0287 (4)	
C17	0.0409 (2)	0.02495 (10)	0.5670 (2)	0.0297 (4)	
H17	−0.0691	0.0385	0.5479	0.036*	
C18	0.1726 (2)	0.06139 (9)	0.51591 (18)	0.0268 (4)	
H18	0.1543	0.1008	0.4618	0.032*	
C19	0.4122 (2)	0.26521 (10)	0.4225 (2)	0.0354 (4)	
H19A	0.3484	0.3065	0.4519	0.053*	
H19B	0.3379	0.2304	0.3835	0.053*	
H19C	0.4941	0.2814	0.3622	0.053*	

### Atomic displacement parameters (Å^2^)

**Table:**

	*U*^11^	*U*^22^	*U*^33^	*U*^12^	*U*^13^	*U*^23^
Cl	0.0366 (2)	0.0513 (3)	0.0393 (3)	−0.01236 (19)	0.0047 (2)	0.0104 (2)
S	0.02092 (18)	0.02869 (18)	0.0272 (2)	−0.00070 (14)	0.00189 (17)	−0.0036 (2)
O1	0.0279 (6)	0.0247 (5)	0.0357 (7)	−0.0001 (5)	0.0022 (6)	0.0033 (6)
O2	0.0221 (5)	0.0340 (6)	0.0447 (9)	0.0030 (5)	0.0019 (6)	−0.0096 (7)
O3	0.0317 (6)	0.0431 (7)	0.0285 (7)	−0.0038 (6)	0.0033 (6)	0.0006 (6)
C1	0.0212 (7)	0.0245 (7)	0.0284 (9)	−0.0012 (6)	0.0030 (7)	−0.0019 (7)
C2	0.0209 (7)	0.0273 (7)	0.0286 (10)	−0.0015 (6)	0.0043 (7)	−0.0023 (7)
C3	0.0298 (8)	0.0244 (8)	0.0335 (10)	0.0010 (6)	0.0024 (8)	0.0002 (7)
C4	0.0305 (8)	0.0300 (8)	0.0310 (10)	0.0027 (7)	0.0008 (8)	0.0011 (8)
C5	0.0246 (8)	0.0346 (9)	0.0314 (10)	−0.0023 (6)	0.0044 (8)	−0.0044 (8)
C6	0.0346 (9)	0.0471 (11)	0.0339 (12)	−0.0016 (8)	0.0003 (9)	−0.0045 (9)
C7	0.0432 (11)	0.0516 (11)	0.0401 (13)	−0.0097 (9)	−0.0022 (10)	−0.0154 (11)
C8	0.0436 (11)	0.0349 (9)	0.0478 (14)	−0.0115 (8)	0.0076 (10)	−0.0133 (9)
C9	0.0347 (9)	0.0287 (8)	0.0406 (11)	−0.0054 (7)	0.0085 (9)	−0.0035 (8)
C10	0.0243 (7)	0.0272 (8)	0.0326 (10)	−0.0025 (6)	0.0054 (8)	−0.0048 (7)
C11	0.0219 (7)	0.0250 (7)	0.0315 (9)	−0.0004 (6)	0.0052 (8)	0.0016 (8)
C12	0.0208 (7)	0.0301 (8)	0.0330 (10)	−0.0023 (6)	0.0029 (7)	0.0009 (8)
C13	0.0227 (7)	0.0257 (7)	0.0247 (9)	−0.0012 (6)	0.0003 (7)	−0.0036 (7)
C14	0.0272 (8)	0.0316 (8)	0.0338 (10)	0.0005 (7)	−0.0081 (8)	0.0026 (8)
C15	0.0373 (9)	0.0339 (9)	0.0330 (11)	−0.0025 (7)	−0.0074 (8)	0.0083 (8)
C16	0.0281 (8)	0.0336 (8)	0.0244 (10)	−0.0071 (7)	0.0010 (8)	−0.0012 (7)
C17	0.0235 (8)	0.0358 (9)	0.0299 (10)	0.0016 (6)	0.0007 (8)	−0.0011 (8)
C18	0.0268 (8)	0.0277 (7)	0.0261 (9)	0.0036 (6)	−0.0008 (7)	0.0001 (7)
C19	0.0321 (9)	0.0337 (9)	0.0403 (12)	0.0032 (7)	−0.0025 (9)	0.0059 (8)

### Geometric parameters (Å, °)

**Table:**

Cl—C16	1.7416 (17)	C7—H7	0.9500
S—O3	1.4397 (16)	C8—C9	1.371 (3)
S—O2	1.4416 (12)	C8—H8	0.9500
S—C1	1.7379 (17)	C9—C10	1.421 (2)
S—C13	1.7663 (17)	C9—H9	0.9500
O1—C12	1.365 (2)	C10—C11	1.409 (3)
O1—C11	1.383 (2)	C12—C19	1.485 (3)
C1—C12	1.366 (2)	C13—C14	1.386 (3)
C1—C2	1.447 (3)	C13—C18	1.394 (2)
C2—C11	1.377 (2)	C14—C15	1.379 (3)
C2—C3	1.418 (3)	C14—H14	0.9500
C3—C4	1.363 (3)	C15—C16	1.396 (3)
C3—H3	0.9500	C15—H15	0.9500
C4—C5	1.440 (2)	C16—C17	1.378 (3)
C4—H4	0.9500	C17—C18	1.382 (3)
C5—C6	1.414 (3)	C17—H17	0.9500
C5—C10	1.415 (3)	C18—H18	0.9500
C6—C7	1.372 (3)	C19—H19A	0.9800
C6—H6	0.9500	C19—H19B	0.9800
C7—C8	1.404 (3)	C19—H19C	0.9800
			
O3—S—O2	119.25 (10)	C11—C10—C5	115.35 (15)
O3—S—C1	108.57 (8)	C11—C10—C9	124.35 (18)
O2—S—C1	106.63 (8)	C5—C10—C9	120.30 (18)
O3—S—C13	107.87 (8)	C2—C11—O1	110.85 (17)
O2—S—C13	107.70 (8)	C2—C11—C10	124.68 (17)
C1—S—C13	106.11 (8)	O1—C11—C10	124.47 (14)
C12—O1—C11	107.04 (13)	O1—C12—C1	109.81 (17)
C12—C1—C2	107.78 (16)	O1—C12—C19	115.45 (14)
C12—C1—S	126.43 (15)	C1—C12—C19	134.68 (18)
C2—C1—S	125.51 (13)	C14—C13—C18	121.29 (16)
C11—C2—C3	119.46 (17)	C14—C13—S	120.70 (13)
C11—C2—C1	104.52 (15)	C18—C13—S	117.99 (13)
C3—C2—C1	136.01 (16)	C15—C14—C13	119.40 (16)
C4—C3—C2	118.21 (16)	C15—C14—H14	120.3
C4—C3—H3	120.9	C13—C14—H14	120.3
C2—C3—H3	120.9	C14—C15—C16	118.81 (17)
C3—C4—C5	122.26 (18)	C14—C15—H15	120.6
C3—C4—H4	118.9	C16—C15—H15	120.6
C5—C4—H4	118.9	C17—C16—C15	122.18 (16)
C6—C5—C10	118.56 (17)	C17—C16—Cl	118.45 (14)
C6—C5—C4	121.40 (19)	C15—C16—Cl	119.36 (15)
C10—C5—C4	120.04 (18)	C16—C17—C18	118.83 (16)
C7—C6—C5	120.1 (2)	C16—C17—H17	120.6
C7—C6—H6	119.9	C18—C17—H17	120.6
C5—C6—H6	119.9	C17—C18—C13	119.47 (16)
C6—C7—C8	121.1 (2)	C17—C18—H18	120.3
C6—C7—H7	119.4	C13—C18—H18	120.3
C8—C7—H7	119.4	C12—C19—H19A	109.5
C9—C8—C7	120.42 (18)	C12—C19—H19B	109.5
C9—C8—H8	119.8	H19A—C19—H19B	109.5
C7—C8—H8	119.8	C12—C19—H19C	109.5
C8—C9—C10	119.4 (2)	H19A—C19—H19C	109.5
C8—C9—H9	120.3	H19B—C19—H19C	109.5
C10—C9—H9	120.3		
			
O3—S—C1—C12	8.85 (18)	C1—C2—C11—C10	−178.97 (16)
O2—S—C1—C12	138.51 (16)	C12—O1—C11—C2	−0.01 (19)
C13—S—C1—C12	−106.87 (16)	C12—O1—C11—C10	179.12 (15)
O3—S—C1—C2	−164.41 (14)	C5—C10—C11—C2	−0.7 (2)
O2—S—C1—C2	−34.74 (17)	C9—C10—C11—C2	179.39 (17)
C13—S—C1—C2	79.88 (16)	C5—C10—C11—O1	−179.69 (16)
C12—C1—C2—C11	−0.25 (19)	C9—C10—C11—O1	0.4 (3)
S—C1—C2—C11	174.06 (13)	C11—O1—C12—C1	−0.16 (19)
C12—C1—C2—C3	−179.16 (19)	C11—O1—C12—C19	−177.64 (15)
S—C1—C2—C3	−4.9 (3)	C2—C1—C12—O1	0.26 (19)
C11—C2—C3—C4	0.1 (2)	S—C1—C12—O1	−173.98 (12)
C1—C2—C3—C4	178.91 (18)	C2—C1—C12—C19	177.06 (19)
C2—C3—C4—C5	0.2 (3)	S—C1—C12—C19	2.8 (3)
C3—C4—C5—C6	179.21 (18)	O3—S—C13—C14	147.32 (16)
C3—C4—C5—C10	−0.7 (3)	O2—S—C13—C14	17.39 (18)
C10—C5—C6—C7	−0.5 (3)	C1—S—C13—C14	−96.49 (16)
C4—C5—C6—C7	179.54 (19)	O3—S—C13—C18	−31.49 (16)
C5—C6—C7—C8	−0.1 (3)	O2—S—C13—C18	−161.42 (14)
C6—C7—C8—C9	0.4 (3)	C1—S—C13—C18	84.70 (16)
C7—C8—C9—C10	0.0 (3)	C18—C13—C14—C15	1.3 (3)
C6—C5—C10—C11	−179.00 (16)	S—C13—C14—C15	−177.48 (16)
C4—C5—C10—C11	0.9 (2)	C13—C14—C15—C16	0.1 (3)
C6—C5—C10—C9	0.9 (3)	C14—C15—C16—C17	−1.1 (3)
C4—C5—C10—C9	−179.14 (17)	C14—C15—C16—Cl	177.77 (16)
C8—C9—C10—C11	179.23 (17)	C15—C16—C17—C18	0.7 (3)
C8—C9—C10—C5	−0.7 (3)	Cl—C16—C17—C18	−178.19 (15)
C3—C2—C11—O1	179.29 (14)	C16—C17—C18—C13	0.7 (3)
C1—C2—C11—O1	0.16 (19)	C14—C13—C18—C17	−1.7 (3)
C3—C2—C11—C10	0.2 (3)	S—C13—C18—C17	177.10 (15)

### Hydrogen-bond geometry (Å, °)

**Table:**

*D*—H···*A*	*D*—H	H···*A*	*D*···*A*	*D*—H···*A*
C19—H19A···Cg3^i^	0.98	2.89	3.488 (3)	120
C8—H8···O2^ii^	0.95	2.48	3.428 (2)	173

Symmetry codes: (i) *x*−1/2, −*y*+1/2, *z*; (ii) −*x*+3/2, *y*+1/2, *z*+1/2.
